# Assessment of glutathione peroxidase enzyme response and total antioxidant status in oral cancer – Systematic review and meta‐analysis

**DOI:** 10.1002/cnr2.1842

**Published:** 2023-06-02

**Authors:** Khadijah Mohideen, Nadeem Jeddy, C. Krithika, Shahul Hameed Faizee, Safal Dhungel, Snehashish Ghosh

**Affiliations:** ^1^ Department of Oral Pathology and Microbiology, Sathyabama Dental College and Hospital Sathyabama Institute of Science and Technology Chennai India; ^2^ Department of Oral Pathology and Microbiology, Thai Moogambigai Dental College and Hospital Dr. M.G.R. Educational and Research Institute Chennai India; ^3^ Meenakshi Academy of Higher Education and Research Chennai Tamil Nadu India; ^4^ Department of Orthodontics and Dentofacial Orthopaedics, Sathyabama Dental College and Hospital Sathyabama Institute of Science and Technology Chennai India; ^5^ Department of Oral and Maxillofacial Surgery College of Medical Sciences Bharatpur Nepal; ^6^ Department of Oral Pathology College of Medical Sciences Bharatpur Nepal

**Keywords:** glutathione peroxidase activity, oral cancer, oral squamous cell carcinoma, total antioxidant status

## Abstract

**Background:**

Oxidative stress induced by free radical accumulation contributes to many pathologies, including cancer. Antioxidant defense system fails to scavenge free radicals when it is excessively accumulated. Assessing individual antioxidant enzymes and total antioxidant capacity could direct the customized therapeutic strategies.

**Objective:**

Evaluation of total antioxidant status and enzyme glutathione peroxidase activity in the oral cancer group compared to the healthy control group.

**Method:**

The literature search included databases PubMed, Science Direct, Wiley Online Library, Cochrane and Cross Reference between 1999 and 2021. The database search was completed in the month of August 2022. The extracted data were analyzed by Comprehensive Meta‐Analysis (CMA) version 3 software (Biostat Inc. Englewood, NJ). Based on search strategies, 1435 articles have been retrieved from the database. In the segregated articles, 1365 were excluded due to duplicated articles, animal studies, low‐quality studies, articles unrelated to the research question, and with unmatched objectives. Based on inclusion criteria, 70 articles were selected for full‐text valuation. However, 33 articles were found highly suitable for inclusion and data extraction. Finally, 11 articles were selected for meta‐analysis.

**Results:**

The meta‐analysis of four included studies of tissue samples showed a significantly (*p* < .001) increased GPx activity in the oral cancer group, when compared to the control group, whereas three included studies of erythrocyte samples displayed a significantly (*p* < .001) decreased GPx activity in the oral cancer group than the control group with the pooled standardized mean difference value of −2.766 moles/min/g Hb at 95% CI (−3.297 to −2.234). The meta‐analysis of the included studies depicted an insignificant (*p* = .947) reduction of salivary TAS levels in the oral cancer group when compared to the control group.

**Conclusion:**

Our systematic review and meta‐analysis depict antioxidant GPx enzyme activity in the regional tissue samples of the oral cancer group differs from other systemic biological fluid samples compared to the healthy control group.

## 1 INTRODUCTION

Oral Squamous cell carcinoma is a primary orofacial cancer arising from the oral epithelium. The reported worldwide incidence is more than four hundred thousand cases annually.^1^

The major etiological factors for OSCC are conventional habits such as smoking or chewing tobacco and consuming alcohol. The interaction of socioeconomic factors, occupational or environmental factors and other local conditions like trauma or sharp teeth, systemic oncogenic viral infections, along with genetic influences such as mutations of oncogenes or tumor suppressor genes (TSG) may play a primary role in oral carcinogenesis.^2^

Traditional habits alike tobacco consumption initiate reactive oxygen species (ROS)/pro‐oxidants formation. ROS are unstable free radicals present with a single unpaired electron in the peripheral shell and are readily reactive. ROS attacks healthy human cells, causing them to lose their normal morphology and function and transmuting them into malignant ones.^3^ ROS includes some radical compounds like superoxide (O_2_^−^) anion, hydroxyl radical (OH^−^), hydroperoxyl radical (HOO^−^) and a non‐radical compound, hydrogen peroxide (H_2_O_2_).^4^ Antioxidants inhibit free radicals' formation and propagation and defend against free radical‐induced injuries. The human body comprises several endogenously developed enzymatic and non‐enzymatic antioxidants that scavenge the reactive species (oxidants), thus being protected from the deleterious effects of ROS.^5^

Superoxide Dismutase (SOD), Catalase (CAT), Glutathione Peroxidase (GPx), reduced Glutathione (GSH), and Glutathione Reductase (GR) are the primary enzymatic antioxidants.^6^ Other antioxidants include ß carotene, vitamins B‐complex, C, and E and the mineral selenium.^7^ These antioxidants are released by the tumor cell or the body's response to tumor growth. When the generation of the reactive oxygen species exceeds the preferred levels due to excessive accumulation/reduced elimination, the consequent deficient level of antioxidants may lead to a disproportion between oxidants and antioxidant defense systems, leading to oxidative stress (OS).^8^ The induced OS results in irreparable cellular/tissue damage, which is vital in cancer initiation, promotion, and progression.

The glutathione antioxidant system includes GPx, GR and Glutathione S‐Transferases (GST). GPx is a prime antioxidant enzyme that helps to remove reactive species once formed. It reduces lipid hydroperoxides to their analogous alcohols. This scavenging antioxidant enzyme is concentrated in the cell membrane and cytoplasm.^9^ In this system, SOD converts superoxide radical (O_2_^−^) into H_2_O_2_; consequently, glutathione peroxidase (GPx) and catalase transform H_2_O_2_ into water.^10^ Therefore, two reactive species, O_2_^−^ and H_2_O_2_, are being converted into the innocuous product water. The induction of the SOD antioxidant enzyme inhibits the superoxide radical reaction on the antioxidant enzyme GPx. It prevents subsequent inactivation of the GPx enzyme and prolongs the activated phase of the GPx enzyme.^7^ The non‐protein thiol, reduced Glutathione (GSH) acts as a cofactor for enzymes such as GPx and glutathione transferase (GST) in the detoxification of lipid peroxides (ROS), thus protecting cells against cytotoxic and carcinogenic chemical reactions during free radicals‐induced oxidative stress.^11^, ^12^ GPx simultaneously catalyzes GSH and form oxidized Glutathione (GSSG) when reacting with free radicals.^13^ Further, GSSG is restored to its reduced form (GSH) by the enzyme glutathione reductase (GR); simultaneously, this reaction maintains a high GSH/GSSG ratio within the cellular system.^14^

Total antioxidant capacity (TAC) measures the free radical‐scavenging ability of the endogenous antioxidant system, comprised of sulfhydryl group (primarily albumin), carotenoids, ascorbate, bilirubin, α‐tocopherol, retinol, urate and some proteins. TAC reflects the tissue's residual antioxidant status after the reactive species' neutralization.^15^

Both endogenous and exogenous antioxidants defend the cellular or local tissue microenvironment from oxidative injury and thus prevent cancer initiation and propagation. The depressed activity of the antioxidant defense system plays a primary role in the development of many pathologies.^16^ Furthermore, tobacco products and areca‐quids were reported to deteriorate the serum level of TAC.^17^ The literature has pointed out that a reduced GSH‐dependent anti‐oxidant system alters the sensitivity of the tumor cells to oxidative or nitrative stress, cytokines, chemotherapeutic drugs and radiation therapy.^18^ So, exploring the link between ROS, the antioxidant system and oral cancer is mandatory. These findings will direct the prospective therapeutic options that target ROS and oxidative stress. Thus, the present systematic review assessed the antioxidant enzyme GPx activity and status of total antioxidants in oral cancer and control group to determine alterations of the antioxidant system in the oral cancer group.

## 2 MATERIALS AND METHODS

The present systematic review (SR) follows the recommended PRISMA protocol. The designated PROSPERO registration number for the present SR is CRD42021271018.

### 2.1 Research question of interest

Are there any differences in antioxidant enzyme GPx activities or levels and TAC values in the oral cancer group compared to the healthy control group?

The PICOS framework for the formulated research query:

- Population: newly diagnosed oral cancer group.
- Interventions: measurement of GPx activities and TAC values (mean ± standard deviation) along with the statistical significance.
- Comparison: compared between the oral cancer group and the healthy control group.
- Outcome: assessment of differences in GPx activity and TAC values between oral cancer and the healthy control group.

### 2.2 Literature search

The literature search was accomplished in electronic databases, such as PubMed, Science Direct, Wiley Online, Cochrane Library and Cross Reference from 1999 to 2021. The literature search was completed in the month of August 2022. The articles in the English language have been selected using the MeSH term and keywords.

### 2.3 Inclusion criteria

- Articles revealed antioxidant status in various biological samples by assessing the activities of enzyme GPx and TAC levels in the oral cancer group and healthy control group.
- Cross‐sectional and case‐control studies conducted in human adults, where filtered by English language and without any restrictions on the sample size.
- The studies included systemically healthy individuals without comorbid conditions or malignancies other than oral carcinoma.
- The studies which included the patients who are not started with therapy for oral cancer and did not use antibiotics, anti‐inflammataries, vitamins or other anti‐oxidant drugs.
- The studies which included systemically healthy individuals who are not under any medications as a control group for comparison.
- Observational studies which stated pre‐therapeutic mean value of GPx activity and TAC values for oral cancer and control groups.
- The studies dividing the oral cancer group into smokers and non‐smokers included only when a healthy control group was formed as a separate evaluation group for the specified marker assessment.
- Studies that provided GPx and TAC mean values or median values with (minimum–maximum) details along with statistical significance *p*‐value for both case and control groups.

### 2.4 Exclusion criteria

- Studies with unrelated objectives or abstracts.
- Duplicated studies involving the same patients by the same authors.
- Being narrated or systematic reviews.
- The studies utilized other antioxidant markers for evaluation.
- The studies have been performed in the oropharyngeal or head and neck cancer group.
- The articles provided insufficient data or graphical representation for comparing the control and oral cancer group.
- The observational studies analyzed the therapy effect without the healthy control group evaluation.
- The studies have not provided adequate data for comparison with other studies.

### 2.5 Assessment of literature

The literature search was performed by three reviewers independently. A consensus was reached if there was disagreement between the reviewers based on specified criteria. The relevancy of the articles was confirmed by analyzing the titles and abstracts of the retrieved articles. The full text was collected for the articles which satisfy the required criteria. Three observers independently examined all the full‐text papers based on the New Castle Ottawa Scale and other limitations such as selection bias, data imprecision, and other research quality measures. After evaluating all the specifics, three authors finally segregated the articles which met the selection criteria.

### 2.6 Data segregation

The examiners analyzed the selected articles, and extracted the data specific to the authors' details, year of publication, sample size, and assessment methodology of GPx antioxidant enzyme activities and TAC values (mean or median) with statistical significance between oral cancer and control group. The values were considered statistically significant if the *p* value was <.05.

### 2.7 Meta‐analysis

The standard difference in the mean was performed for deriving the forest plot to analyze the data with the help of the CMA version 3 Biostatistics software (Englewood, NJ). The pooled mean difference value of GPx activities and the TAC levels between OSCC and the control group was derived at a confidence interval of 95%. The random‐effects model was performed for quantitative synthesis due to the significant heterogeneity of the selected studies. Articles with a similar sample, measurement units and methodology were chosen for the quantitative analysis. Studies that displayed out‐of‐range values were excluded from the meta‐analysis to reduce the chances of heterogeneity.

## 3 RESULTS

**FIGURE 1:**
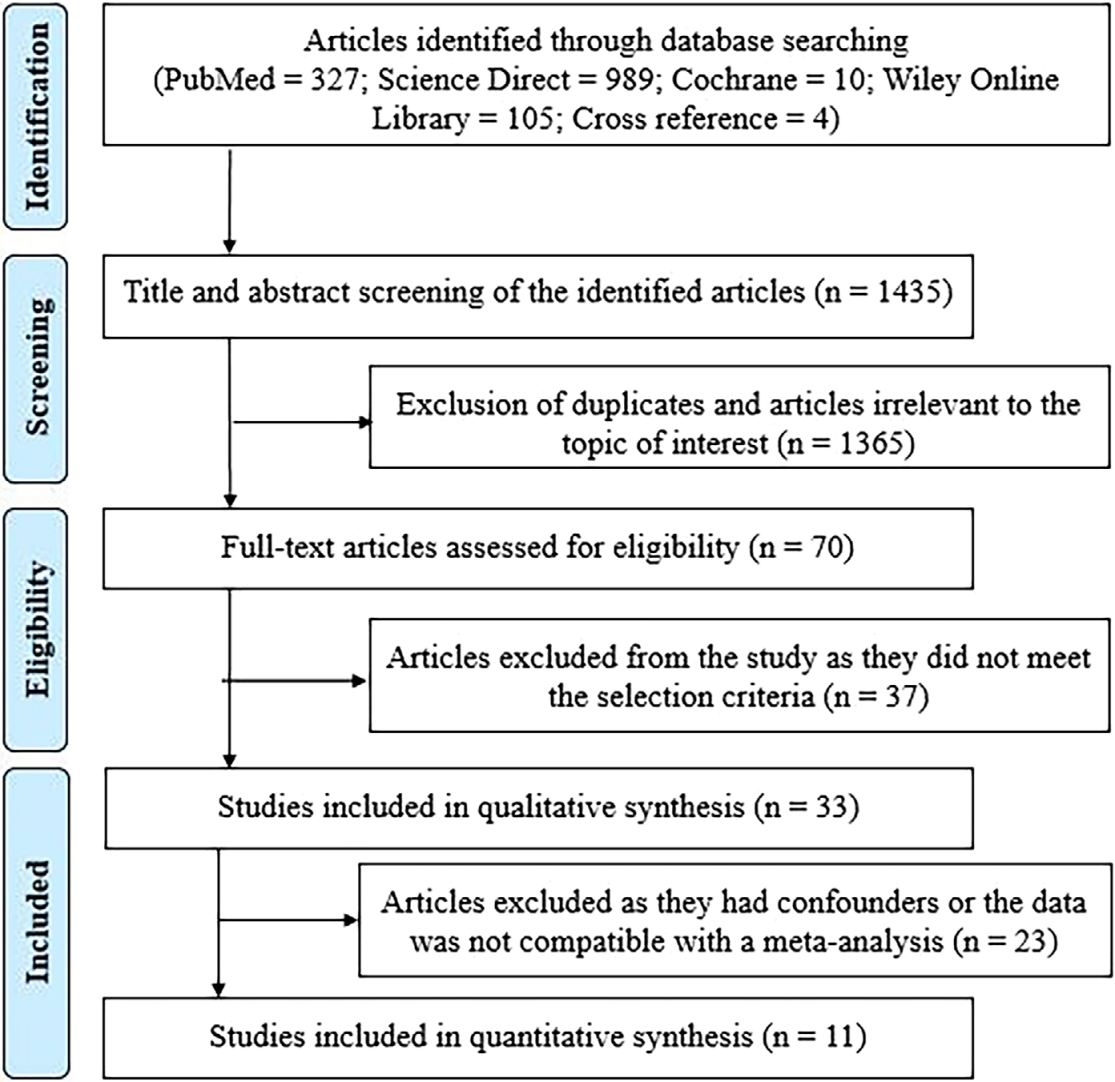
Prisma flow chart explains the selection process of study articles.

From the selected search methodology, 1435 articles were retrieved from search engines. PubMed search provided 327 articles; Science Direct produced 989 papers; Wiley Online library search yielded 105 articles; Cochrane search yielded 10 articles, and cross‐reference pointed to four papers. After search refinement, 1365 articles were excluded because of irrelevancy to the research question of interest and duplication. A full‐text evaluation was done for 70 articles. Upon final screening, the articles with inadequate data (*n* = 3), critical review (*n* = 1), systematic reviews (*n* = 2) and reviews (*n* = 31) were excluded. In article augmentation, 33 articles were found highly suitable for qualitative synthesis. The articles having coherent data and allowed comparison (*n* = 11) were included for meta‐analysis (Figure 1).

The Newcastle‐Ottawa quality evaluation scale was displayed in Table 1 to assess the features of included studies in the systematic review.

**TABLE 1:** New Castle Ottawa Scale for the selected articles.

Study (reference number)	Selection				Comparability		Exposure				Total score
	Case definition	Case representativeness	Control selection	Control definition	Matching known confounding factor	Matching potential confounding factor	Secure patient records	Interviewer blinded to cases and control	Similarity in the case and control ascertainment	Non‐response rate	
Saroja et al.^19^	+	+	+	+	+	−	+	−	+	+	8
Balasenthil et al.^20^	+	+	+	+	+	−	+	−	+	−	7
Subapriya et al.^21^	+	+	+	+	+	+	+	−	+	−	8
Subapriya et al.^22^	+	+	+	+	+	−	+	−	−	+	7
Beevi et al.^23^	+	+	+	+	+	+	+	−	+	−	8
Manoharan et al.^24^	+	+	+	+	+	+	+	−	+	+	9
Khanna et al.^25^	+	+	+	+	+	+	+	−	+	+	9
Fiaschi et al.^26^	+	+	+	+	+	−	+	−	−	+	7
Elango et al.^27^	+	+	+	+	+	+	+	−	+	+	9
Rasheed et al.^28^	+	+	+	+	+	−	+	−	+	+	8
Bahar et al.^29^	+	+	+	+	+	+	−	−	+	−	7
Burlakova et al.^30^	+	+	+	+	+	−	+	−	+	+	8
Arathi et al.^31^	+	+	+	+	+	−	+	−	+	+	8
Srivastava et al.^32^	+	+	+	+	+	−	−	−	+	+	7
Gurudath et al.^33^	+	+	+	+	+	+	+	−	+	+	9
Bagul et al.^34^	+	+	+	+	+	+	+	−	+	+	9
Silpasree et al.^35^	+	+	+	+	+	−	+	−	−	+	7
Manasaveena et al.^36^	+	+	+	+	+	−	+	−	−	−	6
Ganjre et al.^37^	+	+	+	+	+	−	+	−	+	−	7
Thomas and Sethupathy^38^	+	+	+	+	+	−	+	−	−	+	7
Shankarram et al.^39^	+	+	+	+	−	−	+	−	−	+	6
Najafi et al.^40^	+	+	+	+	+	−	+	−	+	−	8
Nyamathi et al.^41^	+	+	+	+	+	−	+	−	+	−	7
Srivastava et al.^42^	+	+	+	+	+	+	+	−	+	+	9
Hamidavi et al.^43^	+	+	+	+	+	+	+	−	+	−	8
Verma et al.^44^	+	+	+	+	+	−	+	−	+	+	8
Khan et al.^45^	+	+	+	+	+	+	+	−	+	+	9
Basu and Guhan^46^	+	+	+	+	+	−	−	−	−	+	6
Subash and Jayanthi^47^	+	+	+	+	+	+	+	−	+	−	8
Deshpande et al.^48^	+	+	+	+	+	+	+	−	+	−	8
Sabarathnam et al.^49^	+	+	+	+	−	−	−	−	+	+	6
Babiuch et al.^50^	+	+	+	+	+	+	+	−	+	+	9
Sushma et al.^51^	+	+	+	+	−	+	+	−	+	−	7

*Note*: The studies that earned scores from 7 to 9 were denoted as high‐quality studies, and no studies scored ≤5, indicating high‐risk studies.

All the segregated data of selected articles were tabulated in Tables 2 and 3. Most studies depicted diminished activity of GPx in OSCC groups in systemic biological samples, whereas local tissue and salivary samples described enhanced GPx activity in OSCC groups than healthy controls (*p* < .05) (Table 2). Similarly, most studies that assessed TAC levels in plasma samples displayed significantly decreased levels in OSCC groups. In contrast, three studies of salivary samples depicted increased TAC levels in OSCC groups compared to the normal control group, which is not statistically significant in the two studies. However, only one study showed declined salivary TAC levels in the OSCC group compared to the normal control group (*p* < .05) (Table 3).

**TABLE 2:** The activities or levels of enzyme glutathione peroxidase in different samples of patients with OSCC and normal control.

Study	Sample	Measurement	Mean OSCC	Std D	Sample size (*n*)	Control mean	Std D	Sample size (*n*)	Stat sig *p* value	Method
Saroja et al.^19^	Tissue	U^a^ g^−1^ Protein	22.79	4.03	33	13.27	3.83	33	<.001	Rotruck et al.^52^
Balasenthil et al.^20^	Tissue	μmol/min/g Protein	21.8	4	10	12.3	3.9	10	<.001	Rotruck et al.^52^
Fiaschi et al.^26^	Tissue	Units/mg Protein	26.91	5.001	18	12.79	2.143	20	<.001	Paglia De and Valentine^53^
Srivastava et al.^42^	Tissue	Units/g Hb	25.79	3.45	20	15.16	0.48	20	.001	Rotruck et al.^52^
Deshpande et al.^48^	Tissue	Units/g Protein	15.96	7.67	20	7.96	2.09	18	<.001	Paglia De and Valentine^53^
Subapriya et al.^21^	Er	moles/min/g Hb	7.54	0.74	24	11.62	1.63	24	<.05	Rotruck et al.^52^
Subapriya et al.^22^	Er	moles/min/g Hb	8.62	0.81	6	11.63	1.12	12	<.05	Rotruck et al.^52^
Beevi et al.^23^	Er	Units/mg Protein	3.33	0.8	15	13.8	1.22	15	<.001	Flohe and Gunzler^14^
Manoharan et al.^24^	Er	Units^a^/g Hb	17.63	1.98	48	22.32	1.86	16	<.001	Rotruck et al.^52^
Rasheed et al.^28^	Er	Units/mg Protein	3.72	1.44	24	9.33	1.61	24	<.001	Flohe and Gunzler^14^
Elango et al.^27^	Er	μmol/min/mg Hb	6.36	0.76	63	9.63	1.2	45	<.001	Rotruck et al.^52^
Nyamathi et al.^41^	Er		12.42	1.42	10	60.46	12.45	10	<.0001	Paglia De and Valentine^53^
Gurudath et al.^33^	Cytosol and Er	Units/g Hb	11.37	1.47	25	45.8	10.32	25	<.0001	Paglia De and Valentine^53^
Manoharan et al.^24^	Plasma	Units^a^/L	189.78	13.4	48	223.84	17.7	16	<.001	Rotruck et al.^52^
Srivastava et al.^32^	Plasma	Units/mg Hb	0.01312	0.00618	20	0.02168	0.00118	20	<.01	Rotruck et al.^52^
Manasaveena et al.^36^	Plasma	μg/dL	13.8		20	65.713		20	<.001	Paglia De and Valentine^53^
Thomas and Sethupathy^38^	Plasma	Units/mL	0.128	0.03	20	0.215	0.04	20	<.05	
Subash and Jayanthi^47^	Plasma	Units/g Hb	14.8	1.7	35	21.8	3.7	30	<.05	Flohe and Gunzler^14^
Fiaschi et al.^26^	Blood	Units/mg Hb	11.41	1.773	18	16.57	2.526	20	<0.001	Paglia De and Valentine^53^
Burlakova et al.^30^	Blood	Units/mg Hb	0.0362	0.0069	50	0.0427	0.0069	54	<.01	Paglia De and Valentine^53^
Sree et al.^35^	Blood	Units/min/mg Protein	7.72	0.93	30	19.71	1.49	30	<.0001	Paglia De and Valentine^53^
Deshpande et al.^48^	Blood	Units/dL	1924.89	421.5	20	1038.51	348.1	18	<.01	Paglia De and Valentine^53^
Khanna et al.^25^	Serum	pmol/mg Protein	0.056	0.022	20	0.134	0.157	20	<.001	Beutler^54^
Bagul et al.^34^	Serum		0.03	0.02	25	0.02	0.02	25	.017	Beutler^55^
Ganjre et al.^37^	Serum		0.0162	0.0174	30	0.0406	0.0338	30	<.01	Spectrometer (Elico)
Khan et al.^45^	Serum	μg/mL	6.51	1.11	50	1.59	0.0037	20	<.001	Aebi^56^
Sushma et al.^51^	Serum	μmols of NADPH oxidized/100 mg protein/h	10.7	0.73	100	13.8	1.25	102	<.005	Lawrence et al.^57^
Shankarram et al.^39^	Saliva	Units/L	95.58	2.46	25	75.04	2.685	25	HS	Competitive ELISA kit (Cayman Chemical Company)
Babiuch et al.^50^	Saliva	Units/L	85.53	22.73	20	90.6	18.65	20	.255	Rotruck et al.^52^
Sabarathinam et al.^49^	Saliva	μg/mg	4.2	0.15	10	2.3	0.25	15	<.05	DTNB Method
Basu and Guhan^46^	Ly	Units/mg Protein	0.04	19	30	0.11	0.27	50	<.001	Beutler^58^

Abbreviations: DTNB, Ellman's reagent (5,5‐dithio‐bis‐2‐nitrobenzoic acid); ELISA, enzyme‐linked immunosorbent assay; Er, erythrocyte; Hb, hemoglobin; HS, highly significant; Ly, lympholysate; OSCC, oral squamous cell carcinoma; Stat sig, statistical significance; Std D, standard deviation.

^a^ μ moles of GSH utilized/min.

**TABLE 3:** Total antioxidant capacity in different samples of patients with OSCC and normal control.

Study	Sample	Measurement	Mean OSCC	Std D	Sample size (*n*)	Control mean	Std D	Sample size (*n*)	Stat sig *p* value	Method
Bahar et al.^29^	Saliva	mmol/L	0.25		25	0.49		25	<.05	ABTS (Randox Kit)
Arathi et al.^31^	Saliva	mmol/L	0.3669	0.3065	25	1.4119	0.3457	25	.001	Koracevic et al. (2001)
Najafi et al.^40^	Saliva		0.101	0.137	22	0.051	0.081	20	.029	
Shankarram et al.^39^	Saliva	mmol/L	1.09	0.08	25	0.91	0.1	25		Competitive ELISA kit
Babiuch et al.^50^	Saliva	mmol/L	0.69	0.44	20	0.51	0.34	20		Benzie and Strain (1996)
Hamidavi et al.^43^	Plasma	mmol/L	0.228	0.0227	40	0.9956	0.0886	35	<.05	Benzie and Strain (1999) (FRAP Assay)
Verma et al.^44^	Plasma	mmol/L	1.06	0.7	20	1.4	0.15	20	.0402	Koracevic et al. (2001)
Subash and Jayanthi^47^	Plasma	μmol/L	616.7	85.6	35	913.8	169.6	30	<.05	FRAP

Abbreviations: ABTS, 2,2′‐azino‐bis(3‐ethylbenzothiazoline‐6‐sulfonic acid); ELISA, enzyme‐linked immunosorbent assay; FRAP, ferric reducing ability of plasma; OSCC, oral squamous cell carcinoma; *p*, probability value; Stat sig, statistical significance; Std D, standard deviation.

Only five studies documented the GPx activities in the OSCC group, considering clinical stages in different biological samples. Two studies pointed out reduction of GPx activities in progressing stage tumors (*p* < .01). However, one study displayed increased GPx enzyme activity in advancing tumors (*p* < .001) (Table 4). The changes concerning clinical T stages and histopathological grades of the OSCC group could not be predicted since only one study reported the specific details (Tables 5 and 6).

**TABLE 4:** The activities of GPx in various samples of patients with different clinical stages of oral cancer.

GPx activity	Study details		OSCC stage II			OSCC stage III			OSCC stage IV			Stat sig	Stage
Author	Sample	Unit	Mean	Std dev	n of cases	Mean	Std dev	n of cases	Mean	Std dev	n of cases	*p* value	Criteria
Manoharan et al.^24^	Er	Units/g Hb	20.04	2.1	16	17.6	2.14	16	15.24	1.71	16	<.01	UICC
Manoharan et al.^24^	Pl	Units/L	204.1	13.73	16	190.15	12.54	16	175.09	13.94	16	<.01	UICC
Srivastava et al.^32^	Pl	Units/mg Hb	13.3	0.41	5	13.47	0.58	7	12.71	0.55	8	NS	TNM
Srivastava et al.^42^	Ti	Units/g Hb	21.82	0.65	5	24.42	1.44	7	29.47	1.32	8	<.001	TNM
Deshpande et al.^48^	Ti	Units/g Protein	‐	‐	‐	16.19	7.68	12	16.43	8.2	8	.26	TNM
Deshpande et al.^48^	Blood	Units/dL	‐	‐	‐	1714.02	1001.2	12	1503.2	920.3	8	.48	TNM

Abbreviations: Er, erythrocyte; n, number of cases; OSCC, oral squamous cell carcinoma; Pl, plasma; Stat sig, statistical significance; Std dev, standard deviation; Ti, tissue; TNM, tumor, node, metastasis; UICC, Union for International Cancer Control.

**TABLE 5:** The activities of GPx and TAC in salivary samples of patients with different T stages of oral cancer.

Study	Salivary sample	Unit	T1 stage (*n* = 4)		T2 stage (*n* = 10)		T3 stage (*n* = 4)		T4 stage (*n* = 2)		Stat sig
			Mean	Std dev	Mean	Std dev	Mean	Std dev	Mean	Std dev	*p* value
Babiuch et al.^50^	GPx	Units/L	81.28	19.39	86.48	28.12	94.9	13.67	70.6	11.6	.672
	TAS	mmol/L	0.63	0.36	0.77	0.57	0.51	0.04	0.78	0.42	.797

Abbreviations: Stat sig, statistical significance; Std dev, standard deviation.

**TABLE 6:** The activities of GPx in various samples of patients with different histopathological grades of oral cancer.

GPx activity	Study details		OSCC (Grade I) *n* = 13		OSCC (Grade II) *n* = 4		OSCC (Grade III) *n* = 3		Stat sig
Author	Sample	Unit	Mean	Std dev	Mean	Std dev	Mean	Std dev	*p* value
Deshpande et al.^48^	Tissue	Units/g Protein	12.75	3.08	16.89	11.11	28.65	2.71	7.18
Deshpande et al.^48^	Blood	Units/dL	1916.09	546.98	1948.63	509.65	1931.39	665.7	.12

Abbreviations: OSCC, oral squamous cell carcinoma; Stat sig, statistical significance; Std dev, standard deviation.

**FIGURE 2:**
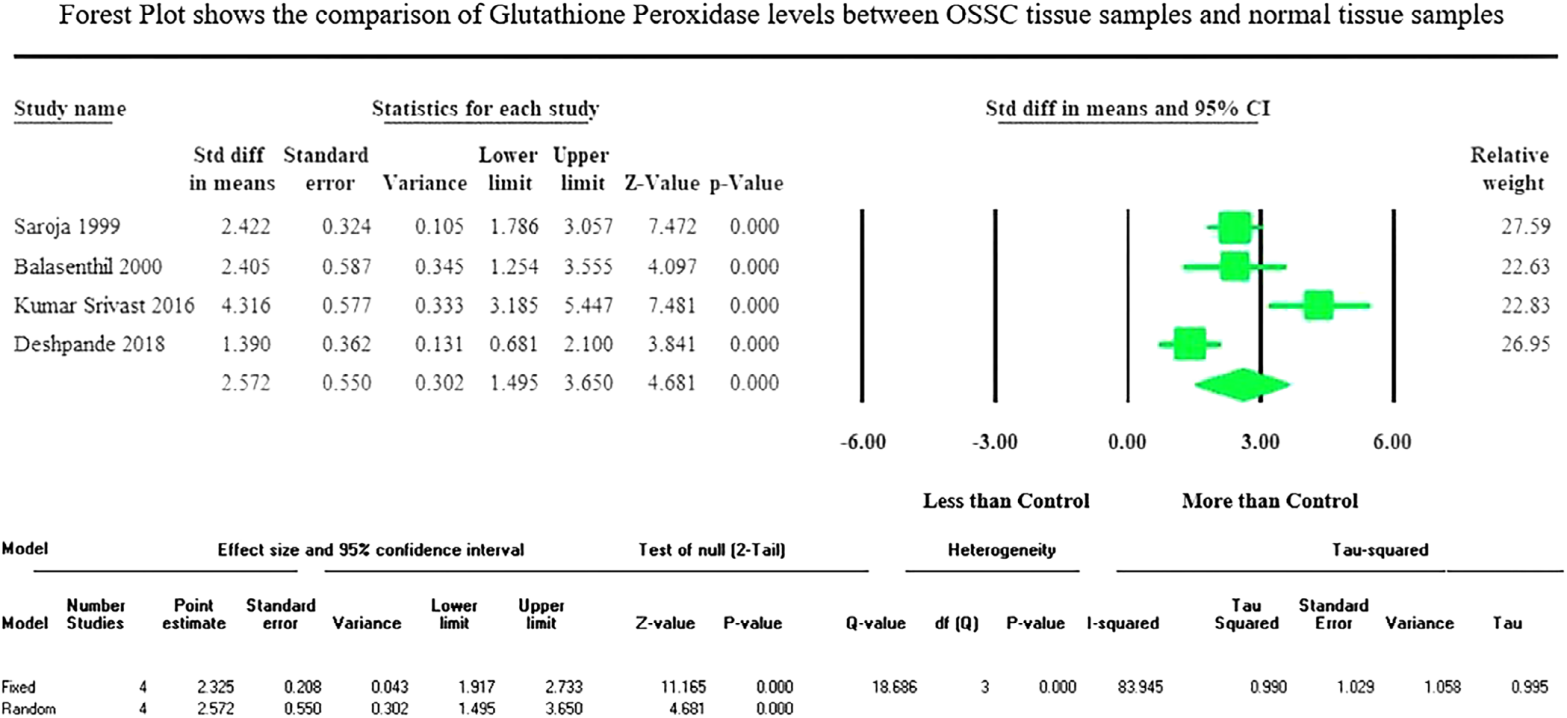
Forest plot expresses the standardized mean difference levels at 95% CI, shows the comparison of glutathione peroxidase activity in tissue samples of OSCC and healthy control group.

**FIGURE 3:**
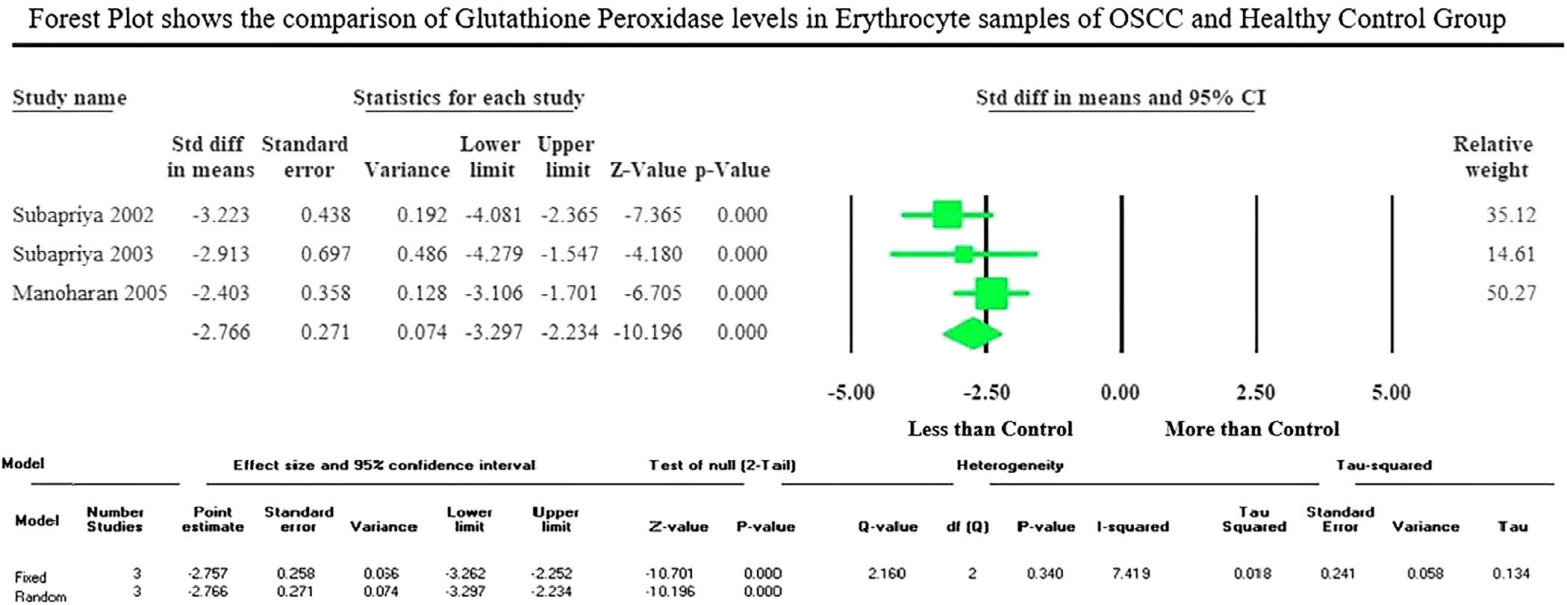
Forest plot expresses the standardized mean difference levels at 95% CI, shows the comparison of glutathione peroxidase activity in erythrocyte samples of OSCC and healthy control group.

**FIGURE 4:**
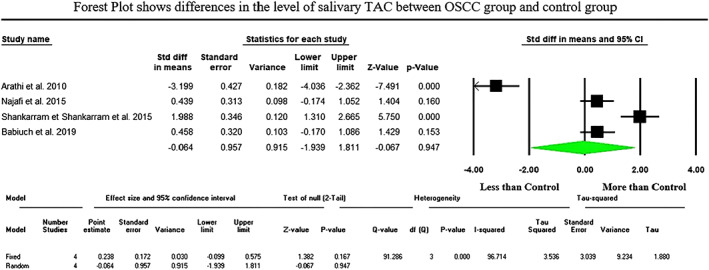
The forest plot displayed the standardized mean difference (Std diff in mean) values at 95% confidence intervals, representing a comparison of salivary levels of total antioxidant capacity between OSCC and normal control groups.

Meta‐analysis results: The meta‐analysis of four included studies of tissue samples showed a significantly (*p* < .001) increased GPx activity in the oral cancer group compared to the control group with the pooled standardized mean difference value of 2.572 μmol/min/g protein at 95% CI (1.495–3.650) (Figure 2), whereas three included studies of erythrocyte samples displayed a significantly (*p* < .001) decreased GPx activity in the oral cancer group than the control group with the pooled standardized mean difference value of −2.766 moles/min/g Hb at 95% CI (−3.297 to −2.234) (Figure 3). The meta‐analysis of the included studies depicted an insignificant (*p* = .947) reduction of salivary TAC level in the oral cancer group compared to the control group, which showed the pooled standardized mean difference value of −0.064 mmoL/L (95% CI −1.939 to 1.811) (Figure 4).

### 3.1 Heterogeneity

The studies taken for meta‐analysis of the activity of GPx enzyme in erythrocyte samples to compare OSCC and normal control group displayed very low heterogeneity, reflected by the *I* ^2^ value 7.419. The studies taken for meta‐analysis of the activity of GPx enzyme in tissue samples and salivary TAC levels to compare OSCC and normal control group displayed high heterogeneity, reflected by the *I* ^2^ values 83.945 and 96.714, respectively (Figures 2, 3, 4). The heterogeneity persisted though the pooled studies showed measurements with reasonably similar methods and excluded the out‐of‐range values. The possible sources of observed heterogeneity could be a lack of standardization in collecting and analyzing samples across studies. In addition, differences in sample characteristics, sample size, study design, or even differences between the populations studied could also contribute to heterogeneity. The meta‐analysis did not permit to perform the sensitivity or subgroup analysis to assess the strength of the findings due to the limited number of studies in each analysis.

Publication bias: A certain asymmetry was perceived on inspecting the funnel plot of the included studies in the meta‐analysis (Supplementary Figures 1–3). Despite the asymmetry of the funnel plot, the present meta‐analysis could have little exposure to publication bias, a finding reinforced by Egger's regression intercept *p* value. The studies included in the GPx assessment of tissue and erythrocyte sample meta‐analysis showed Egger's regression intercept values of 4.988 and −1.615 with two‐tailed *p*‐values of .41 and .68, respectively, indicating a less risk of publication bias in the selected studies. Studies in the salivary TAC assessment meta‐analysis showed Egger's regression intercept value of −31.153 with a two‐tailed *p*‐value of .25, indicating a low risk of publication bias.

## 4 DISCUSSION

Glutathione Peroxidase (GPx) enzyme is a selenocysteine‐dependent antioxidant. The endogenous GPx enzyme is the primary antioxidant that scavenges or neutralizes hydrogen peroxide (H_2_O_2_).^6^ GPx depends on reduced GSH to exert its function. GSH provides reducing equivalents and thus catalyzes the reaction of these enzymes to transform hydrogen peroxide to water.^59^ At the same time, the higher activity of GPx can influence glutathione levels. GPx plays a vital role in detoxification when at a lower concentration of H_2_O_2_, whereas catalase (CAT) becomes active when the GPx pathway attains satiety with a substrate or when there is an excess level of H_2_O_2_.^60^

The present systematic review depicts that there was a significantly enhanced GPx activity in tissue samples^19^, ^20^, ^26^, ^42^, ^48^ and decreased activity in erythrocyte and plasma samples (*p* < .05) of the OSCC group compared to the normal control group.^21^, ^22^, ^23^, ^24^, ^27^, ^28^, ^32^, ^33^, ^36^, ^38^, ^41^, ^47^ Out of the four included studies that analyzed the GPx activity in blood samples except one,^48^ the remaining studies displayed diminished enzyme activity in the OSCC group compared to healthy controls (*p* < .05).^26^, ^30^, ^35^ In consideration of serum samples, three studies displayed reduced activity,^25^, ^37^, ^51^ and the remaining two depicted enhanced GPx activity in the cancerous group compared to the normal control group (*p* < .05).^34^, ^45^ When considering GPx values in salivary samples, two studies displayed intensified activity (*p* < .05)^39^, ^49^; however, one research depicted an insignificant reduction of the GPx activity.^50^ The lympholysate sample described declined GPx enzyme activity in the cancerous group compared to the normal control group (*p* < .05).^46^

The meta‐analysis of four included studies of tissue samples showed a significantly (*p* < .001) increased GPx activity in the oral cancer group compared to the control group with the pooled standardized mean difference value of 2.572 μmol/min/g protein at 95% CI (1.495–3.650), whereas three included studies of erythrocyte samples displayed a significantly (*p* < .001) decreased GPx activity in the oral cancer group than the control group with the pooled standardized mean difference value of −2.766 moles/min/g Hb at 95% CI (−3.297 to −2.234). The meta‐analysis considering the different clinical stages and histopathological grades of the oral cancer group could not be performed due to the scarcity of available reports.

The enhanced glutathione peroxidase activity is due to the higher demand against the toxic substance of the tumor cells and simultaneous defense to protect the prevailing unaffected cells.^61^ According to the previous statement, another study pointed out increased salivary antioxidant levels due to that a compensatory defense reaction.^40^ In contrast, studies have explained that depletion of glutathione peroxidase in overt neoplasias might be due to increased turnover or utilization of antioxidants from the circulation to counteract overwhelming free radicals and resultant oxidative injury.^33^, ^62^ One study correlated low plasma antioxidant levels and tumor burden.^22^ The levels of GPx expression were positively correlated with favorable prognoses, especially the patients with advanced stage IV tumors in one reported work.^63^

The assessment of TAC in various biological samples is a reliable biomarker that comprises the activity of entire enzymatic and non‐enzymatic (vitamins and minerals) antioxidants. It shows higher clinical significance since many pathological conditions alter TAC levels remarkably.^64^

While analyzing the TAC values of salivary samples, three studies displayed augmented level^39^, ^40^, ^50^; however, the remaining two studies displayed depleted TAC levels in OSCC group compared to the control group (*p* < .05).^29^, ^31^ When assessing the plasma samples, three studies depicted reduced TAC values in the OSCC group compared to the normal control group (*p* < .05).^43^, ^44^, ^47^

Few other studies not included in the systematic review displayed significantly decreased TAC levels in the oral cancer group. Korde et al.^16^ found a diminution of TAC in the serum and tissues of patients with oral cancer compared to controls (*p* < .001). Shetty et al.^65^ highlighted that the mean serum TAC value in the oral cancer group was substantially lower than the normal value. Agha‐Hosseini et al. applying the FRAP method, Bahar et al. and Kumar et al. implementing the ABTS method found markedly lower salivary TAC levels in patients with oral or head and neck cancer as compared to healthy individuals.^29^, ^66^, ^67^ Conversely, Wesołowski et al.^68^ utilized the ORAC method and pointed out that mean salivary TAC values were higher in the oropharyngeal cancer group than in the control group (*p* = .029).

The present meta‐analysis of the included studies depicted an insignificant (*p* = .947) reduction of salivary TAC level in the oral cancer group compared to the control group, which showed the pooled standardized mean difference value of −0.064 mmoL/L (95% CI −1.939 to 1.811). The three included studies in plasma samples depicted a significantly (*p* < .05) reduced TAC values in the OSCC group compared to the normal control group.

The reduction of TAC values might be due to the following facts: (1) accentuated utilization of these antioxidants in the scavenging reaction of lipid peroxides and RNS, (2) a poor antioxidant defense reaction in a cancerous microenvironment, (3) an inadequate release of antioxidant enzymes, and (4) increased sequestration of antioxidants by reactive oxygen species. Additionally, it might be caused by a larger magnitude of OS in advanced clinical stages with a higher tumor burden.^16^

The present SR systematically summarized the results of 33 independent studies and pointed out that antioxidant biomarkers GPx enzyme activity and TAC values in peripheral blood of the oral cancer group were lower than in healthy subjects, indicating an elevation of systemic oxidative stress in the oral cancer group. However, GPx response activity in regional biological samples depends upon the local microenvironment of the tumor.

## 5 LIMITATIONS

There was high heterogeneity observed between the included studies. In addition, only a limited number of studies were included in the meta‐analysis, which may impact the generalizability of the findings. Different studies utilized different measurement methods to detect GPx activity and TAC levels, which were reported in different orders of magnitude and affected the level of evidence.

## 6 CONCLUSIONS

Our systematic review and meta‐analysis depict that the regional tissue GPx enzyme activity and the salivary total antioxidant status of the oral cancer group differ from other systemic biological fluid samples of the group compared to the control group. The present meta‐analysis supported the rationale that the imbalance between ROS and the antioxidant system may contribute to the structural and functional remodeling of cells that favor carcinogenesis. Assessing the antioxidant status could contribute to effective intervention and customized therapy. In addition, it helps to determine tumor resistance and monitor the prognosis of patients affected with oral cancer. Regular assessment of antioxidant status may serve as an identification marker for high‐risk group cancer patients. It could be beneficial to decrease the morbidity and mortality of patients with cancer and prolong their survival with better quality of life.

## AUTHOR CONTRIBUTIONS

**Khadijah Mohideen:** Conceptualization (equal); formal analysis (equal); writing – original draft (equal); writing – review and editing (equal). **Nadeem Jeddy:** Conceptualization (equal); methodology (equal); software (equal); writing – original draft (equal). **C. Krithika:** Project administration (equal); supervision (equal); writing – original draft (equal). **Shahul Hameed Faizee:** Formal analysis (equal); writing – original draft (equal); writing – review and editing (equal). **Safal Dhungel:** Methodology (equal); writing – original draft (supporting); writing – review and editing (equal). **Snehashish Ghosh:** Conceptualization (equal); methodology (equal); writing – original draft (equal).

## CONFLICT OF INTEREST STATEMENT

The authors have stated explicitly that there are no conflicts of interest in connection with this article.

## ETHICS STATEMENT

Not applicable.

## Supporting information


**Data S1:** Supporting information

## Data Availability

Data sharing is not applicable to this article as no new data were created or analyzed in this study.
