# Supplementary material for: Assessment of glutathione peroxidase enzyme response and total antioxidant status in oral cancer – Systematic review and meta‐analysis
Source: Cancer Rep (Hoboken). 2023 Jun 2;6(8):e1842. doi: 10.1002/cnr2.1842 (PMC10432471; doi:10.1002/cnr2.1842)
Supplement: Supplementary file 1 — Data S1: Supporting information [file CNR2-6-e1842-s001.docx]

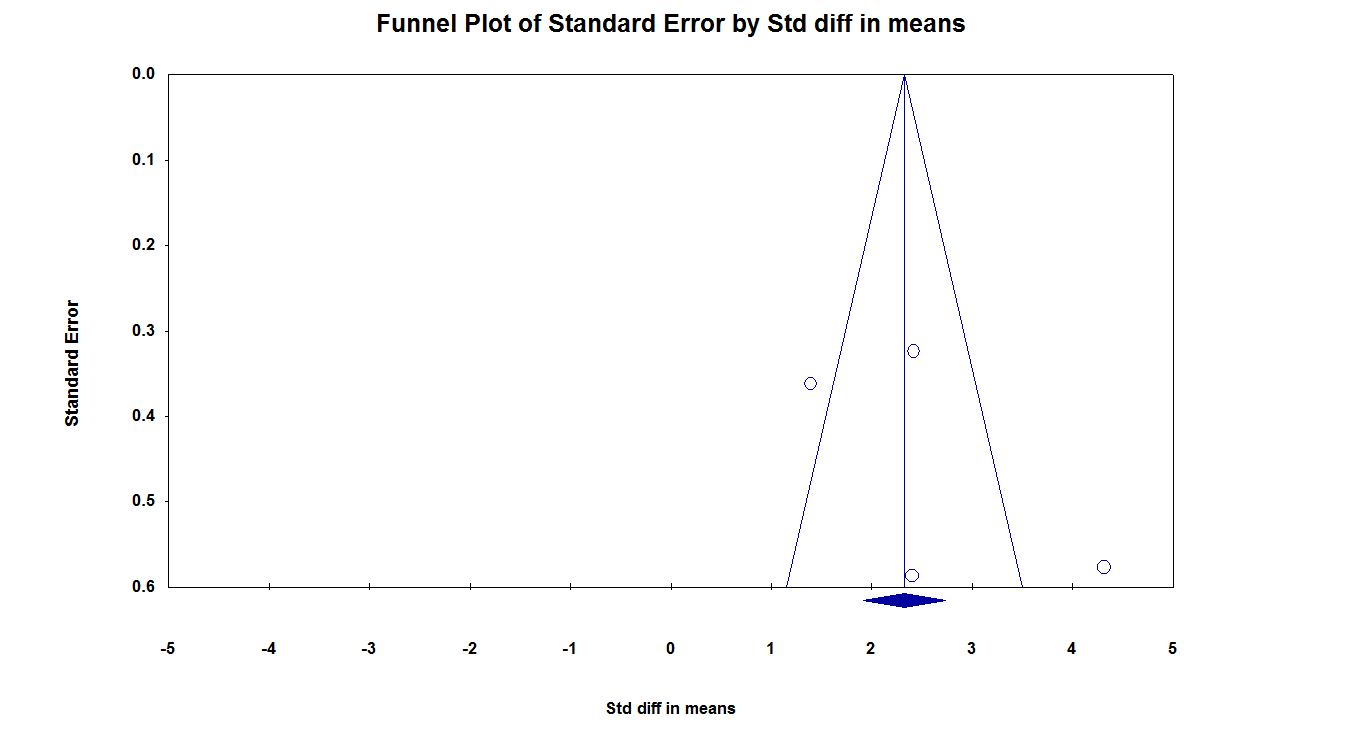


Supplementary Figure 1 - Funnel Plot for included studies in tissue GPx activity assessment meta-analysis


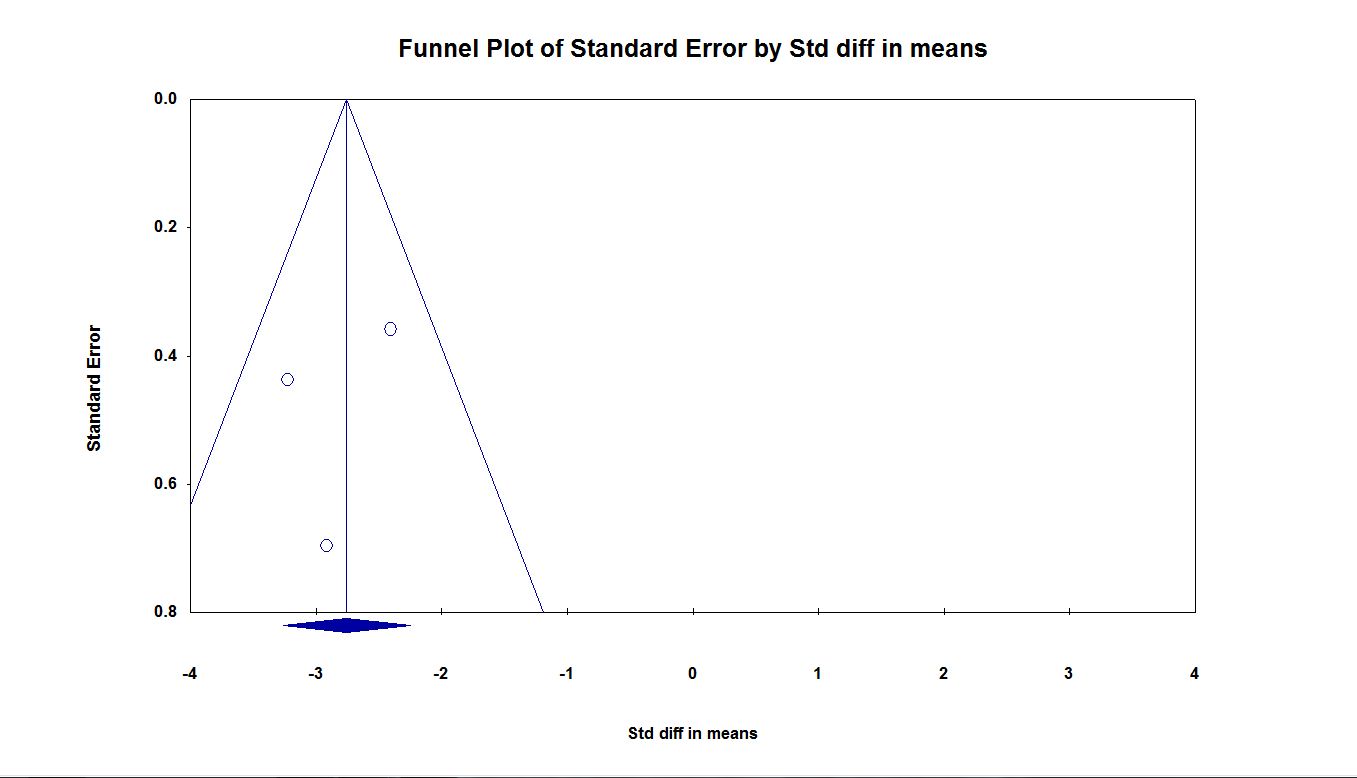


Supplementary Figure 2 - Funnel Plot for Included Studies for assessment of Erythrocyte GPx activity meta-analysis


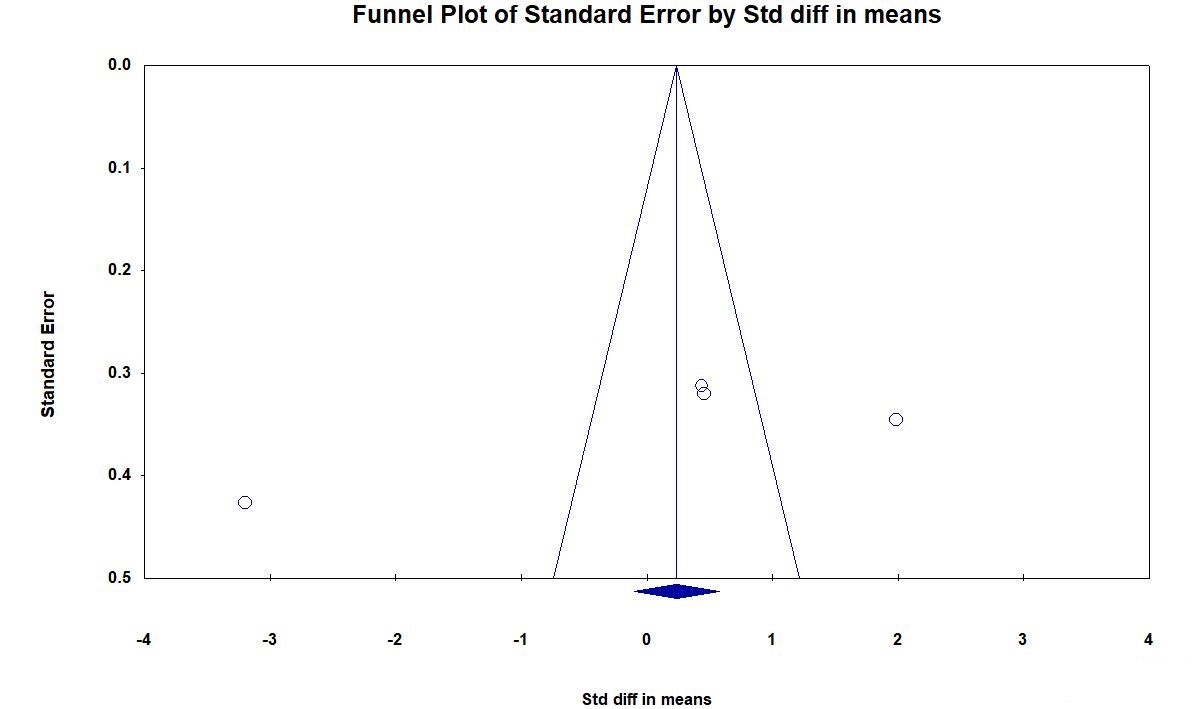


Supplementary Figure 3 - Funnel Plot for studies included in the assessment of salivary TAS level meta-analysis
